# The role of nitric oxide in sepsis-associated kidney injury

**DOI:** 10.1042/BSR20220093

**Published:** 2022-07-11

**Authors:** Filipe Rodolfo Moreira Borges Oliveira, Jamil Assreuy, Regina Sordi

**Affiliations:** Department of Pharmacology, Centre for Biological Sciences, Universidade Federal de Santa Catarina, UFSC, SC, Florianópolis, Brazil

**Keywords:** endothelial nitric oxide synthase, guanylate cyclase, inducible nitric oxide synthase, peroxynitrite, potassium channels, renal haemodynamics

## Abstract

Sepsis is one of the leading causes of acute kidney injury (AKI), and several mechanisms including microcirculatory alterations, oxidative stress, and endothelial cell dysfunction are involved. Nitric oxide (NO) is one of the common elements to all these mechanisms. Although all three nitric oxide synthase (NOS) isoforms are constitutively expressed within the kidneys, they contribute in different ways to nitrergic signaling. While the endothelial (eNOS) and neuronal (nNOS) isoforms are likely to be the main sources of NO under basal conditions and participate in the regulation of renal hemodynamics, the inducible isoform (iNOS) is dramatically increased in conditions such as sepsis. The overexpression of iNOS in the renal cortex causes a shunting of blood to this region, with consequent medullary ischemia in sepsis. Differences in the vascular reactivity among different vascular beds may also help to explain renal failure in this condition. While most of the vessels present vasoplegia and do not respond to vasoconstrictors, renal microcirculation behaves differently from nonrenal vascular beds, displaying similar constrictor responses in control and septic conditions. The selective inhibition of iNOS, without affecting other isoforms, has been described as the ideal scenario. However, iNOS is also constitutively expressed in the kidneys and the NO produced by this isoform is important for immune defense. In this sense, instead of a direct iNOS inhibition, targeting the NO effectors such as guanylate cyclase, potassium channels, peroxynitrite, and S-nitrosothiols, may be a more interesting approach in sepsis-AKI and further investigation is warranted.

## Introduction

It is well known that sepsis is one of the leading causes of acute kidney injury (AKI), compounding an entity called sepsis-induced acute kidney injury or S-AKI. This condition is responsible for 50% of renal dysfunction occurrence in intensive care units (ICUs), making it an important prognostic factor for prolonged permanence and death in the hospital [1]. Worse still, survivors of even a mild form of S-AKI are more prone to develop chronic kidney disease later in life [2], in addition to all other post-septic complications.

Although S-AKI is a well-known sepsis complication, its underlying mechanisms are still poorly understood [1] as are the pathophysiological mechanisms of sepsis. Several mechanisms have been reported as relevant in S-AKI, including microcirculatory alterations [3], oxidative stress [4], and endothelial cell dysfunction [5], to name but a few. One of the common elements to these mechanisms is nitric oxide (NO). Although NO is a potentially relevant player in the pathophysiology of several conditions (sepsis included), the gap in our knowledge concerning the role of NO in S-AKI is wide. A quick search in PubMed revealed 208.510 papers with ‘sepsis’ OR ‘septic shock’ in the Title/Abstract. This number dropped to 3949 when the descriptor ‘kidney injury’ was added. Adding ‘nitric oxide’ OR ‘nitric oxide synthase’ to the search showed 95 and ‘human’ OR ‘man’ only 7 papers. In other words, limited knowledge regarding the role of NO in the S-AKI has been produced so far, and an expressive amount of it has been obtained from animals.

## Nitric oxide system

Due to space limitations, the relevance of NO and NOS will refer only to the kidney in this section. For greater detail on biological chemistry of NO and enzymology of NOS, the reader is referred to some excellent reviews, both classical and recent [6–9].

Soon after the demonstration of the identity between endothelial-derived relaxing factor (EDRF) [10] and NO [11], many reports appeared showing that NO/NOS system was active and relevant for both renal physiology and pathophysiology (see for instance [12–14]).

NO is produced by three NOS isoforms and recently a new biochemical route for NO formation has been described via a reductive ‘nitrate-nitrite-NO’ pathway [15,16]. Whereas the endothelial isoform (eNOS or NOS-3) is found mainly in kidney vessels and glomeruli, interestingly, the neuronal isoform (nNOS or NOS-1) is distributed widely and appears in most of the nephron segments, mainly in cortical structures. The third isoform, the inducible NOS (iNOS or NOS-2) although constitutively expressed in the kidney, appears to be important mainly in inflammatory pathological situations such as S-AKI. Several NO effects in physiology are mediated by its activating effect on soluble guanylate cyclase (sGC), yielding cyclic guanosine 3,5-monophosphate (cGMP), which activates cGMP-dependent protein kinase (PKG), leading to phosphorylation of several downstream targets [17]. However, many NO effects are independent of sGC activation and are carried out by other mechanisms such as post-translational modifications induced by S­nitrosothiols or via peroxynitrite formation, or via the already mentioned ‘nitrate-nitrite-NO’ pathway [13,18,19]. Not only NO affects the kidney but the reverse is true. For instance, the kidney is crucial for L-arginine synthesis from L-citrulline in a way that L-arginine becomes an essential amino acid in renal insufficiency [20].

## Nitric oxide and the kidney

Kidneys present a lower vascular resistance when compared with most organs, and several studies with NOS inhibitors have shown that NO is a key player in the maintenance of the normal vascular tone in the kidney. NO released from the kidney under basal conditions contributes to the control of renal perfusion and influences renal vascular resistance, participating in the regulation of renal hemodynamics [21–25]. Although sodium and water homeostasis are mainly regulated by aldosterone, vasopressin, angiotensin II, and endothelin, NO is also involved in these regulations via different mechanisms. It has been shown that NO participates in the regulation of renal tubular function, inhibiting water and sodium reabsorption along the nephron, and therefore, increasing natriuresis and diuresis [26]. Indeed, deficient renal production of NO in response to increased dietary salt is implicated in the pathogenesis of hypertension [27].

In physiological conditions, the production of NO in the renal medulla exceeds that of the cortex [27–29]. The most consistent finding points a major expression of nNOS in efferent arteriole and in the macula densa, where NO modifies tubuloglomerular feedback [30–33], and, as expected, eNOS is the main isoform in endothelium cells at the renal vasculature [32,33]. The isoform nNOS is activated by increased flow to the macula densa as well as by changes in intracellular pH, contributing to the regulation of afferent arteriole tone and tubuloglomerular feedback [34]. Regarding iNOS, its expression in normal kidney is more controversial. Although it has been shown to be constitutively expressed in this organ [35], iNOS protein and mRNA expression are dramatically increased by stimuli such as lipopolysaccharide (LPS) [36,37], ischemia-and-reperfusion injury [38], and septic [39] and hemorrhagic shocks [40,41].

Acute and chronic nonselective inhibition of the NOS isoforms increase renal vascular resistance, and both nNOS and eNOS isoforms contribute to the control of renal perfusion. However, some studies suggest that the control of the glomerular filtration rate (GFR), as well as the sodium and water homeostasis, is attributed to the NO derived from nNOS, while changes in renal perfusion pressure is attributed to NO derived from eNOS [42]. The differential distribution of each NOS isoform throughout the whole kidney confers a greater level of complexity to the regulation of NO synthesis [30].

The release and the final effect of NO are both modulated by several mediators, such as norepinephrine, angiotensin, vasopressin, endothelin, and reactive oxygen species (ROS) [30]. NO regulates the resistance of afferent arterioles by itself as well as by modulating the actions of other mediators, such as angiotensin II and endothelin. NO significantly modulates the vasoconstrictor action of angiotensin II in preglomerular afferent but not in post glomerular efferent arterioles, contributing to the differential sensitivity to angiotensin II on these vessels, resulting in a greater sensitivity to angiotensin II in the efferent arteriole [43]. Indeed, NO acts in the kidney more as a physiological antagonist of various vasoconstrictors than as a primary vasodilator [44]. An elegant study with isolated and perfused rat kidney showed that a NOS inhibitor causes vasoconstriction in both afferent and efferent arterioles, but the constriction is more intense in the former. According to the authors, NO is only produced in the region of the afferent arteriole or within the glomerulus, and scavengers such as hemoglobin inhibit the luminal transport of NO to the efferent arteriole [25]. It suggests that the differential sensitivities to angiotensin II or even to NO in both arterioles are due to a local NO bioavailability. Moreover, ROS also regulate NO bioavailability and favor vasoconstriction. This reduction in NO supply might be explained, at least in part, because superoxide reacts with NO, forming peroxynitrite, which is a weaker vasodilator than NO [34].

The role of NO in the kidney regulation has been recently reviewed elsewhere [13] and despite some debates, compromised NO bioactivity is associated with kidney diseases and also in S-AKI.

## Sepsis

Sepsis is a life-threatening syndrome characterized by a dysregulated immune response to an infection [45]. The body immune response, allied with the bacterial spread, causes the failure of different systems, culminating in multiple organ damage and death [46]. According to the 2020 report of the World Health Organization (WHO) [47], which reviewed the epidemiological scientific evidence on sepsis, approximately one in every five deaths in the world is caused by sepsis, even though sepsis mortality has decreased by more than 50% in the last 20 years [48]. A great part of the evolution in the sepsis management that led to the reduction in mortality is due to the accumulated knowledge on sepsis pathophysiology. Nowadays, it is well known that the clinical manifestations of sepsis are not related solely to what was first believed to be an increase in immune response or the infectious agent itself; rather, other significant alterations in coagulation, immunosuppression, metabolism, and the function of different systems, such as cardiovascular and urinary, are important aspects that contribute to the sepsis pathological mechanisms [46].

The chain of biological events that induce the onset of sepsis begins with an inflammatory response due to a localized infection. Microorganisms or their constituents—pathogen-associated molecular patterns (PAMPs), such as β-glucans in fungi, lipopolysaccharide (LPS) in bacteria, and the spike protein in the severe acute respiratory syndrome coronavirus type 2 (SARS-CoV-2)—are identified as outsiders by pattern recognition receptors (PRRs) present in cells of the innate immune system [49–51]. In addition, other important signaling sources for the recognition by the innate immune system are the damage-associated molecular patterns (DAMPs). These are intracellular components that, once released into the interstitial space after cell death caused by the microorganism, are recognized by PRRs present in monocytes or macrophages. Thus, the recognition of PAMPs and DAMPs by innate immune cells activates a plethora of pro-inflammatory responses, such as the release of cytokines, activation and proliferation of leukocytes, up-regulation of endothelial adhesion molecules, increased expression of iNOS, and others [52–54]. Usually, all the mentioned biological changes work for the infection depletion and host recovery. To provide an appropriate balance between actions aiming at pathogen clearance and homeostasis and avoiding organic damage, immune cells also release anti-inflammatory cytokines (e.g*.*, IL-10) and other mediators (e.g*.*, prostaglandins) to appropriately control the inflammatory response [55]. However, in sepsis, the initial controlled pro-inflammatory state might be superseded by a prolonged state of dysregulated immunological activity, in which the leukocytes may overreact, causing tissue damage [56], or express fewer chemokine receptors, leading to diminished protective responses and inactivity [57]. Thus sepsis, which was first thought to be an exaggerated pro-inflammatory immune state, is in fact a mix of contradictory responses.

In addition to the immune system, other systems are deeply affected by this dysregulated state. Indeed, the most relevant clinical characteristic of sepsis is the multisystemic failure. Changes at macro and microvascular levels occur and, as result, tissue hypoperfusion sets in. The heart, for example, is greatly affected and cardiac dysfunction is seen in septic patients, culminating in cardiovascular collapse [58]. The release of myocardium depressants [59], mitochondrial damage [60], inefficient calcium dynamics [61], among others, are the main reasons for the cardiac failure. Although this might be the initial event related to the insufficient blood supply to other organs, in some cases, the tissue perfusion deficit might not be necessarily a consequence of the cardiac dysfunction, instead, it would be consequence of a reduction in vascular reactivity [62–65].

Therefore, hypoperfusion comes as one of the main reasons for the tissue biochemical imbalance and organic failure in sepsis [66]. The assessment of the microcirculatory function has gained importance in the clinical management of sepsis [67,68], showing its relevance for sepsis outcomes. One of the major organs most affected by microcirculatory impairment is the kidney. The prevalent paradigm for the kidney injury during sepsis is related to the decreased global renal blood flow and secondary tubular epithelial cell death, or acute tubular necrosis [69]. In the next section, we discuss further the complex mechanisms of the renal flow impairment and acute kidney injury associated with sepsis.

## Sepsis-associated kidney injury (S-AKI)

Sepsis and septic shock are important causes of acute kidney injury (AKI), which is a condition with poor prognostic in hospitals and intensive care units, as renal failure is often a forerunner of multiple organ dysfunction and worsening systemic illness. The real incidence is difficult to estimate, but some studies report that AKI occurs in one in five hospitalized adult patients, which represents almost 50% of adult patients receiving intensive care [70,71].

**Figure 1:**
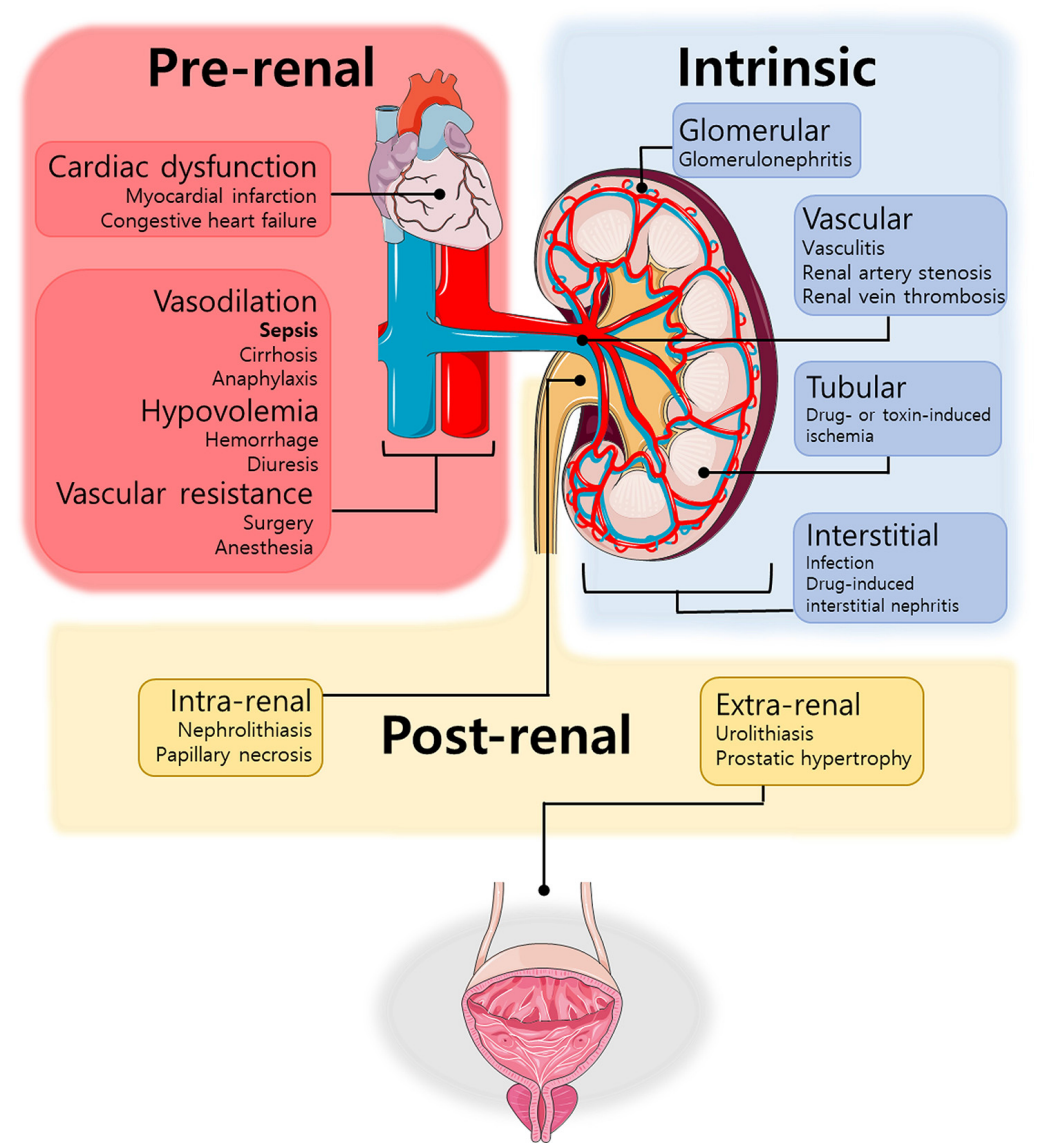
Main causes of renal failure Renal failure is categorized into pre-renal (red), intrinsic (blue), and post-renal (yellow) disease. Pre-renal disease results from reduced blood supply to the renal tissue mainly due to cardiovascular changes, which decreases glomerular filtration rate and, consequently, induces cellular damage. Intrinsic disease is the result of direct cellular injury to the kidney and can be categorized based on the location of the damage (glomerular, vascular, tubular or interstitial). Post-renal disease can be extrarenal or intrarenal and it is usually caused by obstructive uropathy.

Renal diseases can be divided in pre-renal, intrinsic, and post-renal (Figure 1). Septic patients usually develop the pre-renal disease, in which both systemic hypotension and renal vasoconstriction play a role [72]. However, the problem is quite more complex and is not limited to these parameters, as some patients do not present decreased renal blood flow or renal vasoconstriction. Indeed, both renal vasoconstriction and vasodilation have been observed in S-AKI [73]. Several studies show that renal blood flow remained unchanged or even increased in S-AKI. Hypodynamic models characterized by a reduced cardiac output are associated with a reduction in renal blood flow, while models in which hyperdynamic sepsis was produced, renal blood flow increases proportionately to cardiac output. Therefore, S-AKI may occur in a setting of normal or even raised renal blood flow [74].

According to the Kidney Disease Improving Global Outcome (KDIGO), AKI is a subgroup of acute kidney diseases and disorders, and it is classified according to severity and cause, which influence prognosis and management. AKI can be defined by loss of kidney function, increase in serum creatinine level and/or loss of urine production [75,76]. It is associated with increased long-term mortality risk, high mortality rates, and higher costs, especially related to hemodialysis dependency and renal transplant [77]. Electrolyte disorders, volume overload, uremic complications, and drug toxicity are the major complications of AKI. About 30% of critical care patients who survived AKI will require long-term dialysis [72,77].

Although sepsis is the leading cause of AKI, other conditions that also generate AKI might trigger sepsis development. Thus, the sepsis and AKI relationship is complex and inextricably connected in many patients [72]. S-AKI is defined as AKI in the presence of sepsis without other significant factors explaining AKI. As both sepsis and AKI definitions are not too specific, patients that do not necessarily have either of these conditions might be included in S-AKI group. Therefore, the definition of S-AKI comprises a heterogeneous group of diseases. The difficulty in categorizing patients reflects in ineffective treatments associated with high mortality rates [5].

Patients with elevated serum creatinine and low urine output have highest rates of renal replacement therapy, long hospital and ICU stays, and increased mortality [78]. Nonetheless, several limitations restrict the use of serum creatinine and urine output for the diagnosis of AKI. Urine output has an important role for predicting short- and long-term outcomes, but it is difficult to confirm outside the ICU. On the other hand, as muscle perfusion is reduced in sepsis, the production of creatine falls, which blunts the serum creatinine increase, limiting early detection of AKI [79].

Causes and mechanisms of S-AKI are still poorly understood, but there are some hypotheses to explain why sepsis results in AKI. The most acceptable one is the presence of ischemic injury to the kidneys, probably secondary to microcirculatory changes, which can cause redistribution of blood within the kidney, leading to cell damage and to a reduction of glomerular filtration pressure [80,81]. Exacerbated inflammation associated with sepsis is pointed as the main cause of microcirculatory changes, as well as endothelial dysfunction and thrombosis [72,81]. The impairment of the endothelium may reduce the normal effect of eNOS in the kidney to counteract the vasoconstrictive effects of angiotensin II, norepinephrine, and endothelin. Studies with eNOS knockout mice show that lack of eNOS increases renal vascular resistance and blood pressure compared with normal mice, and a small dose of LPS caused a profound decrease in the GFR and renal blood flow in eNOS knockout animals, but not in control mice [82]. A possible explanation for this would be that the decrease in GFR after LPS is caused by the inhibition of eNOS by iNOS-derived NO [83].

Sepsis also affects coagulation and the fibrinolytic cascades and it is considered a procoagulant state that can lead to disseminated intravascular coagulation, which in turn is associated with glomerular microthrombi and consequently, acute renal failure [84]. Studies suggest that there were no consistent relevant histological or immunohistological changes in kidneys affected by S-AKI, thus indicating that it can be a functional phenomenon [85]. Several animal models of S-AKI are used to better understand this complex condition and are summarized on Table 1.

**Table 1:** Animal models of sepsis

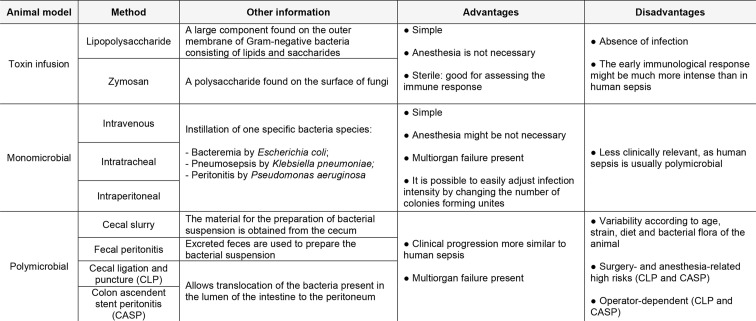

## NO and S-AKI

**Figure 2:**
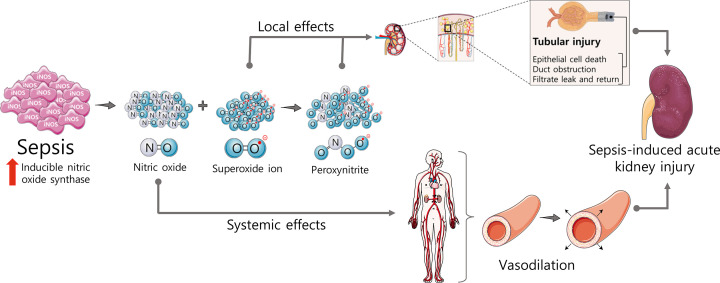
Deleterious effects of nitric oxide (NO) in sepsis-induced acute kidney injury (S-AKI) Sepsis leads the induction of nitric oxide synthase (iNOS) and generation of oxygen radicals, such as superoxide ions, causing peroxynitrite-related tubular injury and NO-induced systemic vasodilation with preserved renal reactivity. Both mechanisms are involved in S-AKI.

Under physiological conditions, the production of NO in the kidneys is low; however, during sepsis, iNOS is predominantly synthesized *de novo*, producing micromolar amounts of NO over prolonged periods. LPS and cytokines stimulate iNOS expression within the kidney, increasing renal tissue NO concentration, which is associated with many deleterious effects [35]. As previously mentioned, NO and other ROS are important regulators of renal function and changes in NO production, localization, and/or bioavailability, together with an increase of oxidative stress, are associated with renal diseases (Figure 2) [86,87].

It has been shown that parenchymal cells, such as the renal epithelium, are the main source of iNOS-derived NO during sepsis, and the contribution of hematopoietic cells, such as monocytes and macrophages, is much smaller in this scenario [88]. As iNOS is constitutively expressed in rat and human proximal tubule cells, a rapid and pronounced response in the kidney is expected during systemic inflammation. NO generation is pointed as the main cause of the subsequent renal hemodynamic changes and reduction of GFR during the first stage of S-AKI [35].

Moreover, despite the increase of NO derived from iNOS in sepsis, the expression of iNOS is not equal in all tissues and this heterogeneity causes variation in regional NO concentrations. In other words, despite the systemic NO levels increase globally, some vascular beds may present a depletion of NO [85]. Even in the same organ, NOS isoforms are differentially expressed, resulting in different amounts of NO locally. For example, even though the production of NO in the renal medulla exceeds that on the cortex physiologically [27,28], it has been shown that iNOS expression in sepsis is more significant in cortex than in renal medulla, causing a shunting of blood from medulla to cortex, potentially leading to medullary ischemia [89]. Medullary hypoxia can be a major driver of cellular and vascular injury, leading to tubular dysfunction. In a model of ovine sepsis, the redistribution of intra-renal blood flow induced by vascular hyperreactivity led to medullary tissue hypoxia, what could be one of the factors leading to S-AKI [90]. It has been shown that the development of renal medullary hypoxia preceded AKI in an ovine model of gram-negative sepsis [80], and the presence of medullary hypoxia was also observed in human patients with S-AKI [91].

Renal failure in sepsis may also be explained by differences in the vascular reactivity among different vascular beds. In general, sepsis studies suggest that vascular resistance and reactivity of different beds are inversely proportional to local production of NO and ROS. While the majority of the vessels are severely dilated and do not respond to vasoconstrictors [92], renal microcirculation behaves differently from nonrenal vascular beds, displaying similar constrictor responses to angiotensin II, norepinephrine, and NOS inhibition in control and septic conditions [93], or even presenting a paradoxically increased vasoconstriction [94]. This renal particularity contrasts with mean arterial pressure and systemic responses, which are usually reduced to vasoconstrictors in different models of sepsis and endotoxemia [95]. From a vascular perspective, this could be a potential explanation for the reduced renal blood flow in sepsis, which leads to S-AKI. Corroborating these findings, a recent study showed that renal vascular reactivity to alpha-1-adrenergic agonist is preserved in sepsis due to the NO-dependent G protein-coupled receptor kinase 2 (GRK2) reduction in the kidneys from septic mice. They also showed that reduction of GRK2 is associated with the preservation of renal alpha-1-adrenergic receptor density and function, which is not observed in other tissues such as the heart, and this fact may contribute for the development of S-AKI [96].

Large amounts of iNOS-derived NO may also inhibit constitutively expressed NOS activity (eNOS and nNOS), resulting in changes of renal microcirculation with the impairment of microvascular homeostasis and renal function [35,83]. The inhibition of eNOS turns the kidney more susceptible to actions of vasoconstrictors, reducing renal blood flow despite increased cardiac output [18,83]. The reduction of eNOS activity is completely prevented by iNOS inhibition *in vitro* and *in vivo* [83]. On the other hand, small amounts of NO are essential for the preservation of GFR in endotoxemic shock, as NO exerts a vasodilatory effect on the afferent arteriole, as well as an antithrombotic effect [97].

Corroborating these findings, studies evaluating therapeutic strategies that increase eNOS expression and/or activity indicated beneficial effects in S-AKI. The up-regulation of eNOS and consequent NO increased production stimulated by the treatment with erythropoietin (EPO) provided beneficial effects in S-AKI induced by cecal ligation and puncture (CLP), with additional improvements in systemic hemodynamic and inflammation [98]. Authors suggested that renal beneficial effect is due to an endothelial protective effect attributable to an EPO-induced increase in eNOS protein expression. Interestingly, another study reported similar findings, showing that blood transfusion is able to improve renal function in rats with S-AKI through restoration of eNOS expression within the kidney [99]. Noteworthy, pharmacological approaches that promote eNOS restoration also reduce renal damage associated with other types of shock [100].

The hypothesis that excessive NO generation is detrimental, whereas basal release of NO is beneficial for the kidney, has stimulated a search for selective inhibitors of iNOS. Of note, nonselective inhibitors of NOS might be detrimental for the kidney as they also inhibit eNOS, while the selective iNOS inhibition has been shown to be protective in S-AKI [35]. A study performed in rats infused with LPS showed that the direct inhibition of iNOS activity can attenuate renal dysfunction through reduction of NO cytotoxic effect, even without any improvement in intrarenal hemodynamics [101]. Several strategies with different iNOS inhibitors in different animal models of S-AKI have been tested and positive results have been seen; however, we still do not have a therapy focusing on iNOS inhibition in the clinical setting. In spite of several data accumulated suggesting beneficial effects of iNOS inhibition, the enthusiasm of clinicians was reduced after a phase III study with a non-selective NOS inhibitor in human sepsis was interrupted before its conclusion [102]. The patient group receiving the NOS inhibitor had mortality rate increased due to cardiac issues. The unfortunate choice of a non-selective NOS inhibitor in this clinical study was translated in many lessons learned, but certainly discouraged new studies with iNOS inhibitors in septic patients. Therefore, the selective iNOS inhibition continues to be a good promise in sepsis and S-AKI but, at least so far, it is still unfruitful.

There is strong evidence that many pharmacological approaches and interventions that cause the indirect inhibition of iNOS activity and/or expression reduce the renal dysfunction associated with sepsis and other types of shock. Inhibition of IκB kinase (IKK) and consequently inhibition of the activation of NF-κB reduced iNOS expression and renal dysfunction associated with septic shock [103], hemorrhagic shock [40], and ischemia–reperfusion injury [104]. A recent study using a dual inhibitor of focal adhesion kinase (FAK) and proline-rich tyrosine kinase 2 (Pyk2) showed iNOS reduction associated with an improvement in renal function in CLP mice [105]. The inhibition of Bruton tyrosine kinase (BTK) has been shown to reduce iNOS and nitrotyrosine immunostaining in immune cells and oxidative stress in renal tissue, resulting in amelioration of renal dysfunction in LPS-induced AKI [106]. Similar findings were found in hemorrhagic shock rats treated with BTK inhibitor [107]. The protective effects of the indirect inhibition of iNOS in S-AKI is very extensive and it has been shown after treatment of septic animals with dexmedetomidine [108] and S-nitrosoglutathione [109], to name but a few. Altogether, these studies suggest that NO has a central role in kidney dysfunction associated not only with sepsis but other severe conditions, such as hemorrhagic shock [41,110] and also ischemia–reperfusion injury [111].

**Figure 3:**
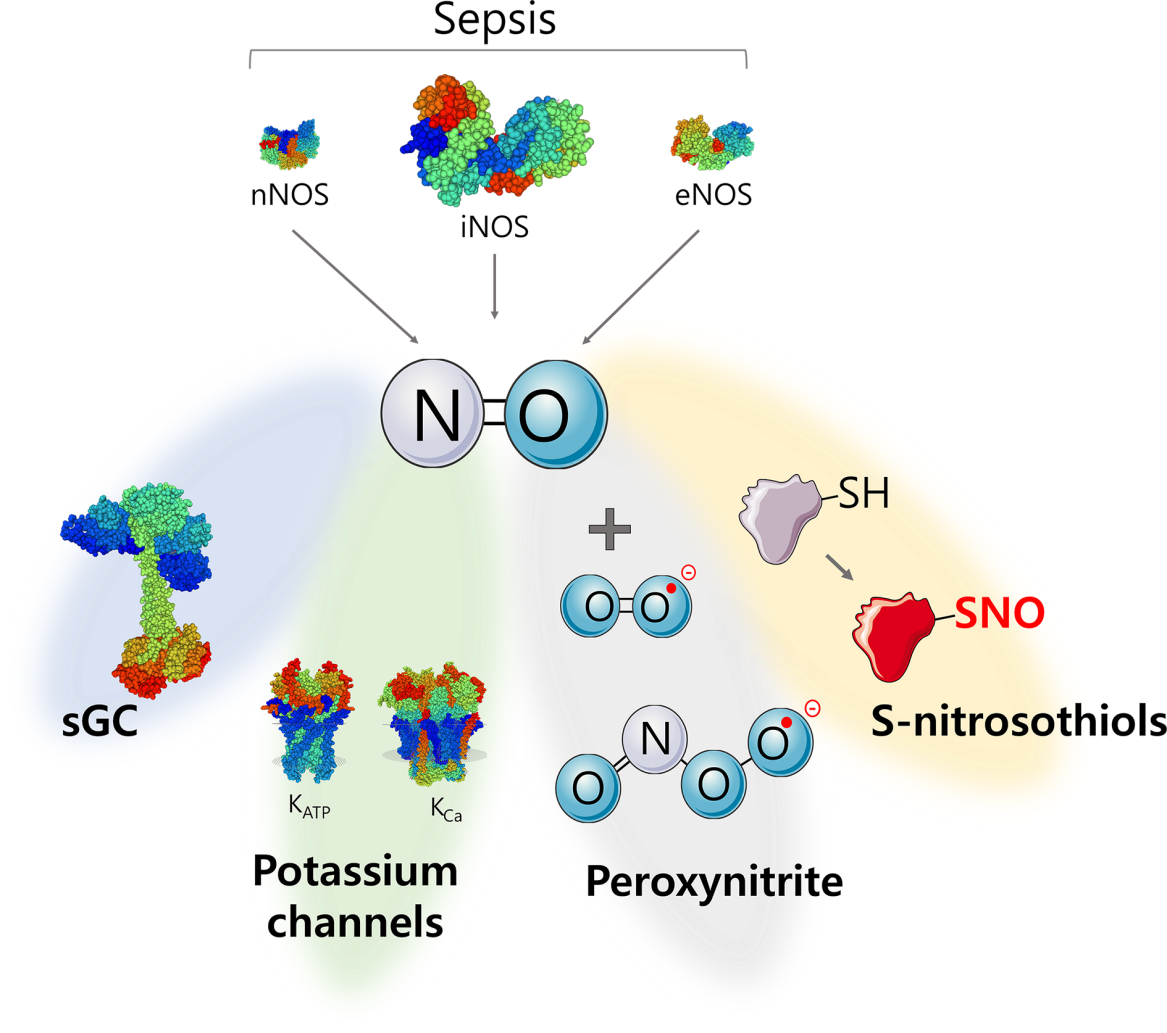
Effector systems activated by nitric oxide (NO) during sepsis NO levels increase during sepsis mainly due to the up-regulation of the inducible nitric oxide synthase (iNOS). There are four main mechanisms elicited by NO: (i) activation of soluble guanylyl cyclase (sGC), which will increase the levels of cyclic guanosine monophosphate; (ii) activation of potassium channels, leading to the increase of potassium efflux; (iii) peroxynitrite formation, culminating in protein nitration; and (iv) S-nitrosylation, by the reaction of the NO with sulfhydryl groups in proteins, which changes protein’s structure and function.

All NO-induced biological changes here described are consequence of the modulation of several targets, such as guanylyl cyclase, potassium channels, peroxynitrite, and S-nitrosothiols (Figure 3). Therefore, the roles of the main NO targets in S-AKI are briefly discussed below.

### Guanylyl cyclase

Most biological actions of NO are mediated by the sGC-cGMP pathway. Soluble GC is a heterodimeric enzyme and upon binding of NO to the heme prosthetic group, cGMP production is increased approximately 200-fold. cGMP interacts with several targets, such as cGMP-activated protein kinases and cyclic nucleotide-gated channels and is hydrolyzed to guanosine monophosphate (GMP) by phosphodiesterases. This pathway is involved in various physiological functions and in several aspects of sepsis vascular dysfunction, and studies are also pointing out the involvement of this system in S-AKI [18,19].

Under pathophysiological conditions, several studies have shown that prolonged oxidative stress deactivates sGC and renders it less responsive to NO [112]. Two main mechanisms have been hypothesized: (i) the oxidative stress may interfere with NO-sGC-cGMP pathway through scavenging of NO and formation of peroxynitrite. Peroxynitrite in turn can oxidize the ferrous iron in the heme prosthetic group of the enzyme sGC which exists in a physiological equilibrium between two redox states, the reduced and oxidized form. The reduced form is NO sensitive, while the oxidized form is inactive and does not respond to NO. The oxidation of ferrous iron can lead to the release of the heme group, turning sGC prone to ubiquitin-mediated proteolytic degradation [113,114]; (ii) a direct oxidation of cysteine thiols to S-nitrosothiols could decrease sensitivity to NO preventing a necessary conformational change or decreasing substrate binding [112,115]. Therefore, under pathophysiological conditions, such as in sepsis, NO-sGC-cGMP signaling may be impaired by, at least, two different mechanisms.

Of note, Knotec and colleagues showed that the desensitization of sGC may occur in the kidney during endotoxemia in mice [116]. The present study showed an increase in serum NO concomitantly with an increase in iNOS in renal cortex. Then, after an increase of cGMP in renal cortical slices 6 and 15 h after LPS injection, cGMP decreased to control level 24 h after the insult. This later decreased cGMP level was not due to an increased degradation, as the use of a phosphodiesterase inhibitor failed to prevent this reduction. Rather, they showed that the catalytic capacity of sGC was diminished at later time points, as the NO donor evoked much smaller production of cGMP in renal cortical slices obtained at 24 h when compared with slices from earlier time points [116]. These interesting findings may help to explain the contradictory renal vasoconstriction despite increased iNOS in the kidneys. Corroborating this finding, it has been shown that there is a NO-dependent reduction of sGC activity after LPS injection in rats. However, in contrast with the kidney tissue, it has been shown that sGC is not functional in lungs and aorta 8 h after LPS administration, but its activity comes back to normal standards 24 h later [117]. In another study, the administration of a sGC inhibitor, methylene blue, reduced kidney plasma markers and decreased mortality rate in late sepsis, but the inhibitor increased mortality when it was administered in early times [118]. In line with this, rats treated with the sGC inhibitor ODQ exhibited renal dysfunction attenuation in both gram-positive (lipoteichoic acid and peptidoglycan) and gram-negative (LPS) sepsis [119], suggesting a crucial role of sGC in S-AKI.

Although the excess of NO and sGC activation/deactivation is associated with kidney injury, the strategic increase of cGMP in specific organs through the inhibition of the enzymes phosphodiesterases proved to be an interesting approach in S-AKI. Administration of sildenafil, a phosphodiesterase-5 inhibitor, was associated with increased renal blood flow and reduced plasma levels of kidney dysfunction markers in early sepsis [120], suggesting a protective role of cGMP in S-AKI.

Altogether, these studies show that NO both activates and inhibits sGC activity (when oxidized) creating a temporal window after LPS administration/sepsis induction in which sGC is not functional, and the lack of functionality is not the same to all organs. This time-dependent dynamics increases the complexity of this condition. Studies evaluating the activity of sGC, as well as cGMP level in different tissues and organs after sepsis induction, are necessary to a better understanding of these findings.

### Potassium channels

Potassium channels, which are important targets for NO, play major roles in renal microcirculation control, and salt and water excretion. Thus, the role of the potassium channels in sepsis pathogenesis has been investigated [121–123]. Although studies designed to investigate the importance of potassium channels in the septic kidneys are not numerous, they suggest an important influence of these channels in S-AKI.

An interesting approach to reduce renal vasoconstriction was performed with the use of ATP-sensitive potassium (K_ATP_) channel opener levosimendan. This drug has potent vasodilator effects, and its administration reduced plasma urea and creatinine concentrations in LPS-treated mice. The levosimendan-mediated protection against LPS-induced AKI was caused by a reduction in renal vascular resistance due to the blockade of angiotensin II constrictor effects [124]. In an ovine model of sepsis, the administration of levosimendan associated with vasopressin and norepinephrine improved renal (and other organs) function when compared with the administration of vasopressin and norepinephrine only [125]. However, despite several small studies have investigated levosimendan in human septic shock and reported an improvement in hemodynamics, microcirculatory flow, and renal and hepatic function, its administration in septic patients failed to reduce organ dysfunction or mortality in the LeoPARDS (Levosimendan for the Prevention of Acute oRgan Dysfunction in Sepsis) trial [126].

Using a different approach, but corroborating these findings, the administration of glibenclamide, a K_ATP_ channel blocker, increased mortality rate and did not show any organ function improvement in CLP rats [127]. This is in line with another study that showed that the blockade of K_ATP_ channels was deleterious for blood perfusion in kidneys, especially when associated with vasoactive drugs which are often used to treat septic vasoplegia. Interestingly, the present study also showed that the reduction in renal blood flow induced by norepinephrine or phenylephrine was even higher in the presence of K_ATP_ channel or calcium-sensitive potassium channel (K_Ca_) blockers, despite the increased blood pressure and vascular reactivity to vasoconstrictors in septic animals [128]. In addition, glibenclamide is a sulphonylurea derivative and aggravates hypoglycemia, limiting its use in shock conditions [127].

In contrast, studies evaluating tetraethylammonium (TEA; a non-selective potassium channel blocker) effects in sepsis suggest a protective, or at least, lack of deleterious effect in the kidneys. The study of Sant’Helena and colleagues [128] showed that intravenous infusion of TEA did not cause acute changes in renal blood flow before or after the administration of vasoconstrictors, thus exhibiting a different profile from glibenclamide. Corroborating these findings, a study that investigated the effects of potassium channel blockers as a possible therapeutic treatment, showed that TEA improved kidney function and reduced mortality in sepsis, while glibenclamide failed to do so [127]. In the present study, CLP animals were treated with potassium channel blockers 4 h after sepsis onset and the effects of the treatment were evaluated 20 h later. Although the effects of the treatment on renal blood flow have not been investigated, evaluation of several inflammatory parameters such as cytokines and iNOS/NO production have shown that injection of TEA caused an overall improvement in septic animals, suggesting a protective effect beyond the blockade of potassium channels on the vasculature. Of note, alterations in the function of potassium channels can cause significant changes in NO production of activated macrophages [129], and this may explain at least in part the reduction of inflammatory parameters observed with TEA treatment, including reduction of iNOS expression and nitrite+nitrate (NOx) levels.

In summary, the studies evaluating potassium channels in S-AKI are sparse, and this seems to be an interesting field to investigate.

### Peroxynitrite

The excess of NO and an increased oxidative stress may also lead to the formation of peroxynitrite through NO reaction with superoxide ions generated during inflammation [130]. Peroxynitrite exerts cytotoxic effects as it acts by oxidizing thiols and DNA bases, and by modifying proteins and lipids through nitration. These damaged cell constituents lead to inhibition of mitochondrial respiratory chain enzymes, DNA damage, and caspases activation. Thus, metabolic stress and cellular dysfunction take place resulting in apoptosis and/or necrosis [131]. Up-regulation of iNOS is associated with proximal tubular injury and peroxynitrite is pointed as the main cause of tubular injury (Figure 2) [132]. Nitrated plasma proteins have been reported in S-AKI patients [133], and nitration of key proteins in cell function and oxidation products are present in the kidneys of LPS [134] and CLP-induced renal injury in rats [135]. It has been shown that resveratrol, a potent scavenger of peroxynitrite, reduced reactive nitrogen species generation, restored perfusion, and protected kidney from septic injury [136]. Corroborating these findings, it has been shown that the extracellular superoxide dismutase maintains renal blood flow in sepsis by decreasing superoxide levels and preventing peroxynitrite generation, therefore, protecting NO bioavailability [137]. Indeed, several strategies aiming to reduce peroxynitrite formation such as N-acetylcysteine, vitamin C and others have been reported in sepsis and revised elsewhere [4,138].

### S-nitrosothiols

Even though protein S-nitrosylation is an important mechanism for many beneficial and harmful effects of NO, this post-translational modification has been less explored in the S-AKI context. The already cited reduction in GRK2 levels in the kidney of septic mice is likely to be due to S-nitrosylation of the enzyme [96]. S-nitrosylation of the transcription factor TonEBP/NFAT5 and the subsequent reduction in its transcriptional activity led to a reduced expression of target genes such as *ClC-K1*, *Barttin*, *urea transporter-A1*, and *aquaporin 2*, all required for urinary concentration. Sepsis-induced urinary concentration defect is related to NO-dependent inactivation of TonEBP/NFAT5, which down-regulates renal medullary solute transport proteins and aquaporin-2 [139]. Finally, protein S-nitrosylation appears to be a relevant target for sepsis (and probably to S-AKI as well) since protein denitrosylation even after the full onset of sepsis proved to be a relevant rescuing factor in septic rats, due to improvements in perfusion and kidney function [140].

## Unanswered questions

Although by no means comprehensive, the following questions point to some relevant lines of research in this field: (a) what is the relative weight of renal vascular dysfunction in S-AKI? (b) what is the main mechanism(s) triggering S-AKI? (c) Is NO protective or harmful to the septic kidney? (d) Is there a time window in S-AKI in which selective iNOS inhibition would be protective? (e) Would phosphodiesterase inhibitors be useful in preserving kidney perfusion during sepsis? (f) What are the relevant mechanisms for chronic kidney disease development in S-AKI survivors?

## Concluding remarks

S-AKI is a very complex condition, and to better understand its pathophysiology, several different animal models of S-AKI are currently being used. However, animal models have several limitations and do not reflect all aspects of human disease. On the other hand, several approaches such as studying the time course of iNOS up-regulation or NO effects on ion channels in renal vasculature are not feasible to be performed in humans. Besides the difference to human sepsis, even the animal models differ from each other. Distinct animal species, models, time-point, all these parameters influence the outcome and conclusions. Therefore, the refinement of animal models of S-AKI is an urgent need.

Several studies show a potential role of NO in S-AKI, and animal studies suggest beneficial effects of iNOS inhibition in sepsis and in S-AKI. However, even three decades later from the initial studies with NO in sepsis, many questions remain. Despite the undeniable involvement of NO in pathophysiology of S-AKI, the inhibition of iNOS as a therapy for human sepsis and/or S-AKI is still unfruitful. The selective inhibition of iNOS keeping eNOS intact has been described as the ideal scenario as NO exerts protective effects that contribute to the maintenance of renal physiology. However, NO from iNOS is important for the immune defense and iNOS is constitutively expressed in the kidneys. Therefore, to inhibit an enzyme, whose product is a mediator that has a dual role—protective and harmful—is quite complex. In this sense, instead of a direct iNOS inhibition, we believe that to target the NO effectors may be a more interesting approach and further investigation on this topic is warranted.

## Abbreviations

AKI: acute kidney injury
BTK: Bruton tyrosine kinase
cGMP: cyclic guanosine 3,5-monophosphate
CLP: cecal ligation and puncture
EDRF: endothelial-derived relaxing factor
FAK: focal adhesion kinase
GC: gualylate cyclase
GMP: guanosine monophosphate
IKK: IκB kinase
iNOS: inducible nitric oxide synthase
K_ATP_: ATP-sensitive potassium channel
K_Ca_: calcium-sensitive potassium channel
NO: nitric oxide
NOx: nitrite + nitrate
Pyk2: proline-rich tyrosine kinase 2
S-AKI: sepsis-induced acute kidney injury
TEA: tetraethylammonium
